# Louisville Summer School of Medicine

**Published:** 1846-01

**Authors:** 


### LOUISVILLE SUMMER SCHOOL OF MEDICINE.

The lectures in the Louisville Summer School of Medicine will commence on the first Monday in April, under, substantially, the arrangements of the last season.

